# Apoptosis and Autophagy: Current Understanding in Tick–Pathogen Interactions

**DOI:** 10.3389/fcimb.2022.784430

**Published:** 2022-01-27

**Authors:** Xin-Ru Wang, Benjamin Cull

**Affiliations:** Department of Entomology, University of Minnesota, St. Paul, MN, United States

**Keywords:** tick, intracellular pathogens, apoptosis, autophagy, cross-talk, *Rickettsia*, *Anaplasma*, *Ehrlichia*

## Abstract

Tick-borne diseases are a significant threat to human and animal health throughout the world. How tick-borne pathogens successfully infect and disseminate in both their vertebrate and invertebrate hosts is only partially understood. Pathogens have evolved several mechanisms to combat host defense systems, and to avoid and modulate host immunity during infection, therefore benefitting their survival and replication. In the host, pathogens trigger responses from innate and adaptive immune systems that recognize and eliminate invaders. Two important innate defenses against pathogens are the programmed cell death pathways of apoptosis and autophagy. This Mini Review surveys the current knowledge of apoptosis and autophagy pathways in tick-pathogen interactions, as well as the strategies evolved by pathogens for their benefit. We then assess the limitations to studying both pathways and discuss their participation in the network of the tick immune system, before highlighting future perspectives in this field. The knowledge gained would significantly enhance our understanding of the defense responses in vector ticks that regulate pathogen infection and burden, and form the foundation for future research to identify novel approaches to the control of tick-borne diseases.

## Introduction

**Figure 1:**
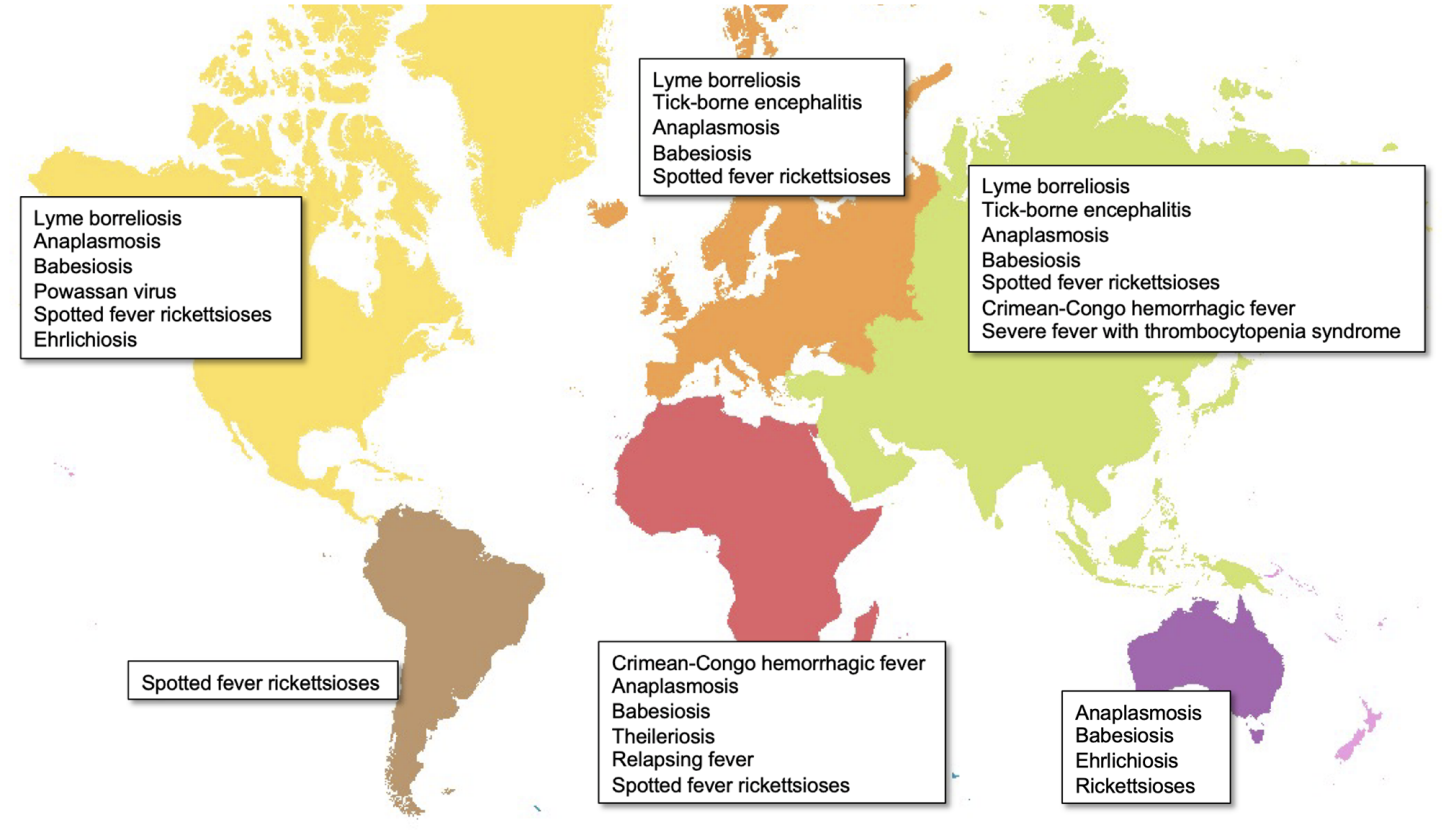
Global distribution of major tick-borne diseases by continent. Map created in ArcGIS online (ESRI, California) using open-source layers from Natural Earth (naturalearthdata.com).

Ticks, as obligate blood-sucking arthropods, can cause substantial public health burdens by direct feeding behaviors and transmitting a broad range of viral, bacterial, and protozoan pathogens to hosts. To date, approximately 80 known tick species are recognized as vectors responsible for spreading emerging infectious diseases throughout the world (**Figure 1**) (Jongejan and Uilenberg, 2004; Ghosh et al., 2007). For example, in the USA, tick-borne diseases (TBDs) accounted for nearly 76.5% of all vector-borne diseases from 2004 to 2016, based on the Centers for Disease Control and Prevention reports (CDC, 2018). Additionally, the effects of human activities and climate change on tick distribution and abundance also increase the risk of emerging and re-emerging diseases (Gray and Banerjee, 1999; Gray et al., 2009). The initial step for the success of intracellular pathogens is survival within their hosts. Tick-borne obligate intracellular pathogens include arboviruses and bacteria, which are responsible for diseases of medical and veterinary importance globally. These pathogens use different strategies to survive within their hosts. For example, after entry into the host cell *Rickettsia* spp. escape the endo-lysosomal pathway and replicate in the cytosol, whilst *Anaplasma* and *Ehrlichia* survive and replicate within specialized vacuoles (Salje, 2021). Considering these diseases are maintained in nature by cycling between ticks and their mammalian hosts, understanding tick-pathogen interactions provides clues for pathogen transmission and establishes a foundation for developing preventative strategies against human infection.

Programmed cell death (PCD) is an essential process in eukaryote homeostasis and development, and includes multiple death programs, such as apoptosis and autophagy (Ameisen, 2002). As a type I PCD mechanism, apoptosis is a genetically regulated process of cellular suicide in multicellular organisms (Taatjes et al., 2008). As a type II PCD mechanism, autophagy is a highly conserved cellular recycling process characterized by lysosomal degradation of cytosol and organelles and recycling of the breakdown products (Levine and Deretic, 2007). Commonly, PCD has been studied in the context of a broad range of human diseases, including neurodegenerative disorders, cancer, and ischemic damage (Elmore, 2007). Recently, different types of PCD have been identified as critical components of innate immunity pathways that act as defense mechanisms against intracellular bacteria, parasites, and viruses, even in insects (Fuchs and Steller, 2011; Romanelli et al., 2014; Koonin and Krupovic, 2019). Like all invertebrates, ticks lack an adaptive immune system but are capable of using the innate immune system to regulate pathogen colonization, persistence, and transmission (Brossard and Wikel, 2004; Hart and Thangamani, 2021). This Mini Review brings together current knowledge on the apoptosis and autophagy pathways from the perspective of tick-pathogen interactions, and our progress in deciphering the mechanisms used by tick-borne pathogens to interact with these pathways. The crosstalk between apoptosis, autophagy and other immune pathways, and how this might be interfered with by intracellular pathogens to benefit their survival are also investigated. Finally, we discuss challenges in studying these pathways in tick-pathogen systems and how these may be overcome in future to improve our understanding of this field and its potential importance to pathogen persistence in ticks and vector competence.

## Apoptosis

As a genetically regulated process of cellular suicide in multicellular organisms, apoptosis responsible for development and homeostasis has been described in several models, including in some arthropods (Jacobson et al., 1997; Bowman and Sauer, 2004; Taatjes et al., 2008; Menze et al., 2010). In vertebrates, apoptosis is also recognized as an innate immune pathway, and mediates eukaryotic cell response to infection by a wide range of pathogens. However, the roles of apoptosis are still perplexing and complex, with multiple pro- and anti-death factors underlying the diversity of events (Everett and McFadden, 1999; Rudel et al., 2010). In some cases, activation of apoptosis is destructive for the pathogens. Pathogens are internalized and packed into the apoptosome during infection, resulting in a more efficient fusion of the phagosome/lysosome, followed by digestion and degradation. Upon apoptosis activation, upregulating nucleases and enzymes can also result in cell demise and promote pathogen clearance (Cervantes et al., 2007; Willingham et al., 2007; Taylor et al., 2008). In addition, efferocytosis, as an antimicrobial effect of apoptosis, allows rapid bacterial killing (Martin et al., 2012; Behar and Briken, 2019). On the other hand, apoptosis can also be advantageous for pathogens. For example, infected apoptotic cells may become unable to contain pathogens and cease to function as a barrier, thus promoting their spread to neighboring cells. Also, in some studies it was shown that induction of apoptosis protected the pathogens against phagocytosis and innate host defenses, which was beneficial for pathogen survival (Green and Kroemer, 2004; Riedl and Salvesen, 2007). Another prominent defense strategy of the host is to eliminate the replicative niche of pathogens *via* the destruction of infected tissues, thereby preventing their replication and dissemination (Labbé and Saleh, 2008). Obviously, the relationships between host and pathogens are complex and involve an intricate balance to serve both host and pathogen interests. This balance of pathogen-induced apoptosis depends on the taxa of bacteria, the duration of infection, the host cell type, multiplicity of infection (MOI), and other factors.

Apoptosis is important for tick development as it initiates salivary gland degeneration, which is regulated by a cascade of caspases leading to the degradation of DNA and proteins in acini (Mao et al., 1995; Mao and Kaufman, 1999; L’Amoreaux et al., 2003; Nunes et al., 2005; Scopinho Furquim et al., 2008). Similar to vertebrates, the regulation of apoptosis in some arthropods and the interactions with pathogens they harbor and transmit have been described (Clarke and Clem, 2003; Flegel, 2007; Sokolova, 2009). However, a few mechanistic insights are just beginning to be revealed in this field due to the wide range of pathogens and their various arthropod hosts. Here, we focus on tick-borne obligate intracellular pathogens to illustrate the paradigms of the function of the apoptotic machinery in ticks.

### Inhibition of Host Apoptosis by Tick-Borne Intracellular Pathogens

A prime example of intracellular bacteria regulating apoptosis is *Anaplasma phagocytophilum*, a tick-transmitted rickettsial agent that causes human granulocytic anaplasmosis. *In vitro*, *A. phagocytophilum* utilizes several mechanisms to inhibit apoptosis in different species of tick cells. For example, besides interfering with endoplasmic reticulum (ER) and the unfolded protein response in *Ixodes scapularis* ISE6 cells, *A. phagocytophilum* also downregulates expression of a series of kinases, including phosphoenolpyruvate carboxykinase (PEPCK), mitogen-activated protein kinase (MKK), and apoptosis signal-regulating kinase 1 (ASK1) (Ayllón et al., 2013; Villar et al., 2015). In addition, in *I. ricinus* IRE/CTVM20 tick cells, transcriptome analysis and flow cytometry revealed that infection with *A. phagocytophilum* not only regulates JAK and anti-apoptotic factors gene expression but also inhibits the intrinsic apoptosis pathway (Alberdi et al., 2016b; Alberdi et al., 2016a). *In vivo*, *A. phagocytophilum* displays a tissue-specific response to mediate cell apoptosis in tick nymphs (Ayllón et al., 2015). It achieves this by targeting the JAK/STAT pathway and decreasing FAS expression in midguts, while reducing porin (voltage-dependent anion-selective channel) expression to inhibit cytochrome c (one of the mitochondrial proteins associated with apoptosis) release from mitochondria in salivary glands *via* intrinsic apoptosis. Interestingly, tick salivary glands serve as an essential organ for *A. phagocytophilum* colonization, and have a possible role in activating extrinsic apoptosis to limit bacterial infection. Indeed, mitochondria, caspases, and pro/anti-apoptotic molecules are key players associated with apoptosis during pathogen infection. Similar to its manipulation of the arthropod host, *A. phagocytophilum* also modulates activity of the above “players” to inhibit apoptosis for their advantage in vertebrate hosts (Scaife et al., 2003; Borjesson et al., 2005; Choi et al., 2005; Ge and Rikihisa, 2006; Niu et al., 2010).

Another very similar system is exploited by *Rickettsia rickettsii* (agent of Rocky Mountain spotted fever). According to the proteome of *Rhipicephalus microplus* BME26 cells, *R. rickettsii* may hamper apoptosis *via* inhibition of caspase-3 activity, thus favoring bacterial growth and proliferation (Martins et al., 2020). Apart from bacterial pathogens, tick-borne viruses have long received much attention. The *I. ricinus* IRE/CTVM20 tick cell transcriptome exerts different gene expression patterns relative to infection with *A. phagocytophilum* after tick-borne encephalitis virus (TBEV) and louping ill virus (LIV) infection, such as raising cytochrome c expression. Interestingly, some apoptosis-related genes, including caspase and hsp70, are expressed differently in flavivirus and intracellular bacterial infections. Whether flaviviruses could benefit from inhibiting tick apoptosis as does *A. phagocytophilum*, is however unconfirmed (Mansfield et al., 2017).

### Activation of Host Apoptosis by Tick-Borne Intracellular Pathogens

Although a critical strategy of intracellular pathogens is induction of vertebrate host-cell apoptosis under certain circumstances, the pro-apoptotic response of ticks to tick-borne pathogens is much less defined. Recently, we have shown that *Rickettsia parkeri*, a tick-transmitted spotted fever group rickettsia, is able to activate mitochondria-dependent apoptosis to promote its infection of and replication in tick cells (Wang X-R. et al., 2021). By employing cell types from different tick species, we demonstrated that *R. parkeri* initiation of apoptosis was a conserved response and required intracellular rickettsial replication. Considering that apoptosis is initiated *via* a series of stressors within a cell, the different growth status (exposure, colonization, invasion, and infection) of pathogens in the host might cause different stimuli, thus playing a diverse role in apoptosis. Indeed, *R. parkeri* exerts pro-and anti-apoptotic activities during different infection phases, which also has been observed in another spotted fever group rickettsia, *R. rickettsii* (Clifton et al., 1998). However, unlike in the arthropod host, *R. parkeri* failed to induce apoptosis in vertebrate host cells at the same infection phase (Wang X-R. et al., 2021). Unsurprisingly, pro-apoptotic activity in one cell type or host species may not be the same as in another cell type or host species. Another example of a pathogen that induces different responses in mammalian and tick hosts is Hazara virus, a tick-borne segmented negative-sense RNA virus closely related to Crimean-Congo hemorrhagic fever virus (CCHFV), but which causes less severe disease. Cleavage of virus nucleocapsid (N) protein by caspase-3 results in apoptosis in mammalian cells but fails to activate apoptosis in tick cells. The use of two different strategies to modulate apoptosis in the respective hosts by members of the genus Nairovirus could directly affect the infection outcome (Fuller et al., 2019). Obviously, a flexible strategy is important for the successful colonization of vectors and hosts by those pathogens in a broad range of host cell types and host species. However, how intracellular pathogens execute this flexible strategy is largely unexplored in their arthropod hosts.

## Autophagy

Autophagy is an important eukaryotic homeostatic pathway involved in the recycling of intracellular constituents and survival during starvation. Although various subtypes of autophagy exist, the best understood is macroautophagy (often simply called autophagy), characterized by formation of a double membrane-bound vesicle called an autophagosome and subsequent trafficking of the autophagosome to the lysosome for breakdown of its contents (Feng et al., 2014). Compared to our rapidly advancing knowledge of autophagy in vertebrates, that of autophagic processes in ticks is still in its infancy, with the core autophagic machinery only identified in a few species (Umemiya et al., 2007; Kawano et al., 2011; Flores Fernández et al., 2014; Umemiya-Shirafuji et al., 2014; Flores Fernández et al., 2016; Moura-Martiniano et al., 2017; Wang et al., 2020). As well as its important role in starvation (Umemiya-Shirafuji et al., 2010; Umemiya-Shirafuji et al., 2014; Moura-Martiniano et al., 2017; González Castillo et al., 2019; Rosendale et al., 2019; Wang et al., 2020), autophagy in ticks is also involved in embryo development (Umemiya-Shirafuji et al., 2010; Kawano et al., 2011; Umemiya-Shirafuji et al., 2014; Flores Fernández et al., 2014; González Castillo et al., 2019) and degeneration of salivary glands (Yu et al., 2017; Wang et al., 2018; Wang Y. et al., 2021), in which apoptosis also plays a role. Despite autophagy being a key component of host innate immune response to pathogens [known as xenophagy (Wang and Li, 2020)], the function and mechanism of autophagy in the interactions between ticks and tick-borne pathogens is completely unknown. Considering that autophagy is a highly conserved process, the common strategies employed by tick-borne pathogens in vertebrates, such as manipulating the host autophagic machinery to evade engulfment and destruction in the lysosome, and/or to direct autophagic processes, may also apply in ticks. Here, we center on the Rickettsiales (Patterson et al., 2021; Salje, 2021; Voss and Rahman, 2021), to display the autophagy mechanisms exploited by tick-borne intracellular bacteria.

### Subversion/Evasion of Host Autophagy by Tick-Borne Intracellular Bacteria

The most well-studied mechanisms used by tick-borne bacteria to manipulate the autophagic pathway come from the Anaplasmataceae in their interactions with mammalian cells. *Anaplasma phagocytophilum* secretes the effector protein Ats-1, which interacts with Beclin1 to recruit autophagosomes to the *A. phagocytophilum* vacuole, supplying nutrients to support pathogen growth (Niu et al., 2012). A similar but distinct process to induce autophagy and trafficking of autophagosomes to the pathogen-containing vacuole is employed by *Ehrlichia chaffeensis* (causative agent of human ehrlichiosis) *via* its effector Etf-1, which interacts with Rab5, Beclin1 and the autophagy-initiating class III phosphatidylinositol 3-kinase complex (Lin et al., 2016). Both *A. phagocytophilum* and *E. chaffeensis* also prevent their vacuoles fusing with the lysosome (Niu et al., 2008; Lin et al., 2016; Lina et al., 2017); this is achieved by *E. chaffeensis* through modulation of the Wnt signaling pathway to inhibit autolysosome formation (Lina et al., 2017).

Infection with a range of spotted fever group rickettsiae (*R. conorii, R. japonica, R. montanensis, R. parkeri* and *R. rickettsii*) results in autophagy induction in mammalian host cells (Uchiyama et al., 2012; Engström et al., 2019; Sahni et al., 2020). Pathogenic rickettsiae appear to be capable of evading this immune response, whilst non-pathogenic species lack this ability (Uchiyama et al., 2012; Engström et al., 2019). Infection of ﻿human umbilical vein endothelial cells with *R. rickettsii* or *R. conorii* results in mTOR activation, potentially as a mechanism by which these rickettsiae limit anti-microbial autophagy (Sahni et al., 2020), whilst *Rickettsia parkeri* is able to evade autophagy by employing outer membrane protein B (OmpB) to prevent the ubiquitination of surface proteins and their subsequent recognition by autophagic receptors in both ﻿human microvascular endothelial cells and mouse bone-marrow-derived macrophages (Engström et al., 2019). Even in the same cell type, different *Rickettsia* species utilize contrasting strategies to evade autophagy. For example, *R. australis* induces autophagy to aid successful invasion of mouse bone-marrow-derived macrophages (Bechelli et al., 2019), resulting in the inhibition of inflammatory cytokine secretion to favor bacterial survival (Bechelli et al., 2021). Due to the broad range of rickettsial pathogens and different host cell types studied, we are just unveiling the tip of the iceberg regarding the complex interactions between these pathogens and their hosts. Although we can use the situation in vertebrates as a basis for predicting what might occur in ticks, we cannot assume that the strategies employed by pathogens to infect mammals can be generalized to their persistence in arthropods (as shown above with apoptosis), and so further investigation into how pathogens interact with tick autophagy are warranted.

## Discussion

### Limitations of Apoptosis and Autophagy Study in Tick–Pathogen Interactions

**Figure 2:**
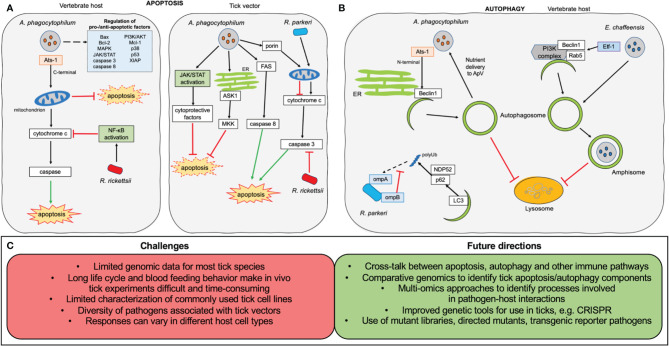
Overview of apoptosis and autophagy research on tick-borne obligate intracellular pathogens. **(A)** Manipulation of apoptosis. In the vertebrate host (left) the C-terminal fragment of the *Anaplasma phagocytophilum* effector Ats-1 localizes to the host mitochondria where it inhibits apoptosis. A large number of kinases and pro-/anti-apoptotic factors (shown in pale blue box) also show changes in expression during *A. phagocytophilum* infection. *Rickettsia rickettsii* infection leads to NF-κB activation, inhibiting cytochrome c release from mitochondria resulting in downstream inhibition of apoptosis. In the tick vector (right), *A. phagocytophilum* uses multiple pathways to inhibit apoptosis, including activation of the JAK/STAT pathway, down-regulation of mitogen-activated protein kinase (MKK) and apoptosis signal-regulating kinase 1 (ASK1), decreasing FAS expression, and reducing porin expression to inhibit cytochrome c release from the mitochondria. *Rickettsia rickettsii* prevents caspase 3 activation in order to inhibit apoptosis. *Rickettsia parkeri* infection is associated with increased mitochondrial cytochrome c release, leading to increased activation of apoptosis, which is essential to its infection of tick cells. **(B)** Manipulation of autophagy. Experimental evidence only exists from mammalian systems. The N-terminal portion of the *A. phagocytophilum* effector Ats-1 localizes to the endoplasmic reticulum (ER) where it interacts with Beclin1 to initiate autophagosome formation. Autophagosomes are prevented from trafficking to the lysosome and instead fuse with the bacterial vacuole to deliver membrane and nutrients to *A. phagocytophilum.* The Etf-1 effector secreted by *Erhlichia chaffeensis* interacts with Beclin1, Rab5 and the PI3K complex to induce autophagosome formation. The autophagosomes fuse with the *Erhlichia-*containing vacuole and are prevented from fusing with the lysosome by bacterial interference with the Wnt signalling pathway. During *R. parkeri* infection of macrophages, ompB shields the rickettsial surface protein ompA from polyubiquitination, preventing its recognition by the autophagy adaptors p62 and NDP52. **(C)** Challenges and future directions in the research of interactions of intracellular tick-borne pathogens with apoptosis and autophagy pathways.

Despite impressive progress being made on how pathogens “tamper with” the tick immune system, including revealing antibacterial and antiviral pathways and identifying molecular effectors and cells (Fogaça et al., 2021), unlike the well-known arthropod-pathogen systems (Buchon et al., 2014), tick-pathogen interaction is a newly emerging field that is far from completely understood. This is largely due to the tick’s own complex development and the diversity of its transmitted pathogens. Firstly, *in vitro* study depends on tick cell lines, which are relatively fragile compared to more common cell types, and have more intensive culture requirements (Munderloh and Kurtti, 1989; Bell-Sakyi, 1991; Mattila et al., 2007; Bell-Sakyi et al., 2009). Additionally, most tick cell lines were derived from embryos, however, their tissue(s) of origin are unconfirmed. Different cell types might possess different characteristics and ontogenies, resulting in unique properties (Mattila et al., 2007; Wang et al., 2020). *In vivo* studies on ticks are more challenging because of their unique life cycle, which is influenced by an array of elements, including species, host feeding preference, different ecological and geographic factors in nature, and strict maintenance requirements in the laboratory (Sonenshine and Roe, 2013; Jia et al., 2020). Besides some medically and veterinary important species, most ticks are a blind-spot due to insufficient data on genomic information, let alone the interactions with potential pathogens that they may harbor. Genomic data is only available for a limited number of tick species including *Ixodes scapularis, Ixodes ricinus, Ixodes persulcatus, Haemaphysalis longicornis, Dermacentor silvarum, Hyalomma asiaticum, Rhipicephalus sanguineus*, and *Rhipicephalus microplus* (Gulia-Nuss et al., 2016; Cramaro et al., 2017; Jia et al., 2020). The most critical difficulty is pathogens themselves, as many different factors such as species/strain pathogenicity, difficulty in laboratory maintenance, etc. may alter the observed results. For example, even different strains of the same pathogen, *R. rickettsii*, exhibits exclusive manners in different cell types or organs (Lehman et al., 2018). Thus, pathogens initiate a critical step for tick immune response, directing more complex communication than simple inhibition or activation. Studies to address these difficulties would pave the way for future research centered on the tick immune system (**Figure 2**).

### Future Perspectives of Apoptosis and Autophagy Study in Tick–Pathogens Interaction

It is unquestionable that PCD acts as one piece of the puzzle for tick innate immunity, and more work needs to be done to gain more clues to solve a tick’s “Jigsaw puzzles”. This would include investigating the network of other immune pathways as well as the cross-talk between apoptosis and autophagy under certain conditions (Fairlie et al., 2020). Paradigms in vertebrate hosts utilize these communications to enhance the recognition and destruction of intracellular pathogens (Hua et al., 2019; Van Opdenbosch and Lamkanfi, 2019), and we expect that immune responses in ticks behave similarly. Interestingly, tick-borne intracellular pathogens also make use of effective communications in their vertebrate hosts to tip the scales in their favor. For example, as well as inducing autophagy, both the *A. phagocytophilum* effector Ats-1 and the *E. chaffeensis* Etf-1 are also translocated into the host mitochondria to inhibit apoptosis initiation (Niu et al., 2010; Liu et al., 2012). Another *A. phagocytophilum* effector AptA induces autophagy and the ubiquitin-proteasome system, whilst reducing the efficiency of apoptosis (Ma et al., 2021). There is also interplay between autophagy and inflammatory pathways during both *Ehrlichia* and *Rickettsia* infection (Tominello et al., 2019; Bechelli et al., 2021), and autophagy induction is balanced by signaling of MyD88 (a downstream adaptor for many pattern recognition receptors) during ehrlichial infection (Kader et al., 2017).

As with other arthropods, crosstalk within the tick innate immune system associated with the response to pathogen infection has also been explored in recent decades (Capelli-Peixoto et al., 2017; Fogaça et al., 2021). Although antibacterial and antiviral pathways, including JAK-STAT (﻿Janus kinase/signal transducer and activator of transcription), Toll, IMD (Immune Deficiency) and RNA interference (RNAi), possess a certain specificity, they are also capable of collaboration under certain conditions. For example, ticks utilize the IMD pathway in response to infection with *B. burgdorferi*, *A. phagocytophilum*, or *A. marginale* (Shaw et al., 2017; McClure Carroll et al., 2019; Kurokawa et al., 2020). The downstream pathways include NF-kB/Relish and Jun N-terminal kinase (JNK) (Ramphul et al., 2015; Chowdhury et al., 2020; Tafesh-Edwards and Eleftherianos, 2020), which are involved in viral-induced apoptosis and have been well characterized in insects. It is possible that bacteria induce a similar response in ticks. However, the components of these pathways in ticks are highly divergent from vertebrate and insect systems, and whether those mechanisms also apply in ticks needs further investigation. Towards this end, identification of the apoptosis and autophagy components of ticks by employing comparative genomics would be a significant step. Utilizing tick and other arthropod genome sequences, the homologues to apoptosis/autophagy-related genes of known function should be confirmed (Wang et al., 2020).

To understand the biological processes involved in apoptosis/autophagy in response to pathogens, multi-omics (including genetics, epigenetics, transcriptomics, proteomics, metabolomics, and cellomics) would be valuable approaches (Hasin et al., 2017; Chu et al., 2021). For example, combining genomic and transcriptome data can reveal apoptosis-related genes and their roles in pathogen infection. Using metabolomics and proteomics, proteins involved in the apoptosis pathway and their connection to molecular changes in metabolic pathways can also be identified. Single-cell/nucleus omics also can determine functional molecules of each cell and specific tick cell subtypes in response to pathogens. Finally, combining properly analyzed approaches and apoptosis assay measurements, the study of apoptosis in tick-pathogen interactions would be significantly enhanced. Genetic tools for editing microorganisms, including mutagenesis and CRISPR as well as RNAi, facilitate the growing body of research in host-pathogen interactions (Durvasula et al., 1997; Billmyre et al., 2013; Wilke and Marrelli, 2015; Vo et al., 2021). As well as using the multi-omics methods outlined above, further work to characterize interactions between pathogens and tick autophagy could employ transgenic reporter bacteria or viruses to visualize responses to autophagy. In addition, with the use of directed mutants or random mutant libraries, the underlying mechanisms used by pathogens to influence autophagy and apoptosis pathways could be revealed, as well as identification of specific factors essential for bacterial invasion and replication in their tick vectors (**Figure 2**).

## Summary

Characterization of the involvement of the tick PCD machinery in pathogen acquisition, persistence, and transmission would help explain the natural cycle of tick-borne pathogens, as well as lead to the design of specific targets for new vaccines and drugs to prevent or treat TBDs. Greater knowledge of tick-borne intracellular pathogen and host (both ticks and mammals) interplay could have implications for understanding how the innate immune system contributes to the vector competence of various tick species for different intracellular pathogens and to the ability of vertebrate hosts to act as reservoirs or succumb to disease.

## Author Contributions

X-RW and BC conceived and wrote the article. All authors contributed to the article and approved the submitted version.

## Funding

The study was financially supported by a grant to UGM from the NIH (2R01AI049424), and a grant to UGM from the Minnesota Agricultural Experiment Station (MIN-17-078).

## Conflict of Interest

The authors declare that the research was conducted in the absence of any commercial or financial relationships that could be construed as a potential conflict of interest.

## Publisher’s Note

All claims expressed in this article are solely those of the authors and do not necessarily represent those of their affiliated organizations, or those of the publisher, the editors and the reviewers. Any product that may be evaluated in this article, or claim that may be made by its manufacturer, is not guaranteed or endorsed by the publisher.
